# Complementary Feeding in the Preterm Infants: Summary of Available Macronutrient Intakes and Requirements

**DOI:** 10.3390/nu12123696

**Published:** 2020-11-30

**Authors:** Guglielmo Salvatori, Ludovica Martini, the Study Group on Neonatal Nutrition and Gastroenterology—Italian Society of Neonatology

**Affiliations:** Neonatal Nutritional Education and Human Milk Bank Unit, Department of Medical and Surgical Neonatology, “Bambino Gesù” Children’s Hospital, IRCCS, 00165 Rome, Italy; ludovica_martini@yahoo.it

**Keywords:** complementary feeding, weaning, preterm infants, macronutrients

## Abstract

Limited data are available regarding the nutritional needs for preterm infants. In most cases, guidelines refer to the acquisition of neuromotor skills, adequate weight and corrected chronological age. While waiting for the establishment of specific nutritional indications for premature infants we proposed the weaning recommendations for term infants of the Italian Society of Human Nutrition with LARNs (Reference intake Levels of Nutrients and energy for the Italian population) of 2014, the Dietary Reference Values for nutrients of European Food Safety Authority (EFSA) of 2017 and the Nutrient Reference Values for Australia and New Zealand Including Recommended Dietary Intakes of 2017.

## 1. Introduction

Complementary feeding (CF), is defined by the World Health Organization (WHO) in 2002, as that process that starts when breast milk alone is no longer sufficient to satisfy the nutritional requirements of infants so that the addition of other foods and liquids is necessary [1]. The inclusion of infant formulas as CF has the goal of promoting and encouraging breastfeeding [2]. For infants at term, both the American Academy of Pediatrics (AAP) and the WHO suggest exclusive breast feeding for the first 6 months with the goal of optimizing somatic growth, neurological development, and overall health [3,4]. Similarly, the European Society for Pediatric Gastroenterology, Hepatology and Nutrition (ESPGHAN) recommends delaying the introduction of CF until at least 17 weeks of age, but no later than 26 weeks, therefore recommending exclusive breastfeeding until six months [2]. Nevertheless, these recommendations are not conceived for preterm infants.

The WHO defines ”preterm” babies born before 37 weeks of pregnancy, and divides the preterm population into “extremely preterm” (<28 weeks), “very preterm” (28 to <32 weeks) and “moderate to late preterm” (32 to <37 weeks) [5].

It is now well knows that fetal and infancy growth rates have profound long term consequences on health both in term and preterm infants [6] and may be influenced by diet [7,8] and age of introduction of solid foods [9].

In most cases, guidelines for weaning preterm infants refer to the acquisition of neuromotor skills, adequate weight and corrected chronological age and no evidence-based guidelines are currently available on optimization of the type and sequence of foods to be provided to for preterm infants.

In 1994 the Committee on Medical Aspects of Food Policy (COMA) included, in the guidelines on weaning, a section on preterm infants which included recommendations to determine the appropriate time to start weaning. They advised to initiate the weaning process when the infant has reached at least 5 kg of weight has no longer tongue thrust reflex, is able to eat from a spoon and the weaning diet is able to assure adequate nutrition [10].

The Joint Consensus Statement on weaning of preterm infants dated 2008 suggested that in preterm infants weaning should be started between five and eight months of uncorrected age to ensure that sensitive periods for the acceptance of solid foods are not missed and to allow development of the appropriate feeding skills [11].

While waiting for the establishment of specific nutritional indications for premature infants, a valid option is represented by the weaning recommendations for term infants.

## 2. Nutritional Recommendations for Term Infants

The Italian Society of Human Nutrition with LARNs (Reference intake Levels of Nutrients and energy for the Italian population) of 2014, the Dietary Reference Values for nutrients of European Food Safety Authority (EFSA) of 2017 and the Nutrient Reference Values for Australia and New Zealand Including Recommended Dietary Intakes of 2017, can all be used as references [12,13,14].

Dietary reference values include [12,13,14]:

- Population Reference Intakes (PRI): the amount of a nutrient that must be consumed on a regular basis to maintain health in an otherwise healthy population.
- Average Requirements (AR): a daily nutrient level estimated to meet the requirements of half the healthy individuals. This assumes that the caloric intake and all other nutrients are already satisfied.
- Adequate Intake (AI): is the value estimated when a PRI cannot be established. An AI is the average daily level of intake by a population group of apparently healthy people that is assumed to be adequate.
- Reference Intake ranges for macronutrients (RI): intake range for macronutrients, expressed as % of the total energy intake. These apply a wide range of intakes that are adequate for maintaining health and are associated with a low risk of selected chronic diseases.
- Tolerable Upper intake Level (UL): highest average daily chronic nutrient intake level likely to pose no adverse health effects to almost all individuals in the general population.
- Suggested Dietary Target for the population (SDT): amount of a nutrient required to prevent or reduce the risk of chronic disease.

### 2.1. Water

Water is an essential nutrient. It is necessary for protein and glycogen synthesis and it is required for thermoregulation, digestion, absorption, dissolution of nutrients and elimination of waste products (Table 1). It includes water from beverages of all kind.

The AI is calculated assuming a daily breast milk intake of 600 mL This supplies 0.52 L water/day. An amount of 0.32 L is adjuncted from complementary foods to reach a total daily amount of 0.84 L rounded to 0.8 L [13].

### 2.2. Protein (Maintenance and Growth)

Proteins have functional and structural properties. Proteins are made by 20 amino acids, and constitute many hormones, enzymes and coenzymes. Nine amino acids are considered “essential” meaning they cannot be produced by the body, and must be obtained from food sources: histidine, isoleucine, leucine, lysine, methionine, phenylalanine, threonine, tryptophan and valine. In case of preterm birth or during catabolic illnesses, other non-essential amino acids (arginine, cysteine, glutamine, glycine, proline and tyrosine) may also be demand in the diet and are termed “conditionally indispensable”. Alanine, aspartic acid, asparagine, glutamic acid and serine are non-essential [14]. The I total amount of proteins (Table 2, Table 3 and Table 4), their digestibility and their composition (richness of essential amino acids) must be considered. Foods of animal origin with a high protein content include meat, fish, eggs, milk and dairy products. High-protein plant foods include bread, other grain-based products and leguminous vegetables. Most of the animal sources are high quality proteins having a high content of indispensable amino acids, while plant proteins usually have a lower content [13].

AI is calculated by multiplying the breast milk protein concentration (11 g/L) by the volume of breast milk (0.6 L) and adjuncting a compensation for complementary foods of 7.1 g/day. The digestibility and comparative protein quality of formulas must be accounted for as these will be different to human milk [14].

### 2.3. Carbohydrates and Dietary Fiber

The primary function of carbohydrate is providing energy. Given that limited data exist to estimate dietary requirement for carbohydrates and dietary fiber it is difficult to set an AR, RI or AI (Table 5, Table 6 and Table 7).

The type of carbohydrate consumed has an impact on health outcomes. Carbohydrates can be divided in “glycemic carbohydrates”, digested and absorbed in the small intestine and “dietary fiber”, not digestible, which transit into the large intestine. The main glycemic carbohydrates are monosaccharides, disaccharides, malto-oligosaccharides and starch. The term “sugars” is often used to indicate monosaccharides and disaccharides. The term “added sugars” generally refers to sucrose, fructose, glucose, starch hydrolysates (glucose syrup, fructose syrup) and other sugar preparations used alone or included during food preparation. Reference values for carbohydrate intake must also consider other energy delivering macronutrients and is generally expressed as percentage of the total energy intake (En%). While evidence exists that >20 En% of sugars can increase serum triglycerides and cholesterol and that >20–25 En% can negatively influence the insulin response, the currently available data are not sufficient to define an exact upper limit intake of added sugar. Diets rich in fat and low in carbohydrates are associated with adverse effects on body weight, although the evidence is insufficient to define a lower intake threshold [12].

### 2.4. Fats

Fats are a significant source of energy and energy requirements remain high throughout the first year of life. A low-fat diet will typically result in a diet with a low energy density, which may mean that the total amount of food needed to meet energy requirements is so large that the infant is unable to eat enough [2]. Most of the fats in foods are triglycerides (a unit of glycerol and three fatty acids). Other dietary fats include phospholipids, phytosterols and cholesterol. Fatty acids are one of the major energy sources for the body but are also involved in many other vital processes (structural components of cell membranes, precursors for important molecules, regulation of enzyme activities). There are three major kinds of fatty acids: saturated, monounsaturated and polyunsaturated.

Saturated fatty acids do not have double bond and are found in milk, cheese, butter, most animal meats, palm and coconut oil, and often in biscuits, cakes and pastries. They can be synthesized by the body and are therefore not absolutely required in a diet. Monounsaturated fatty acids have one double bond. The main one is oleic acid. They are contained in olive and peanut oils and are also not required in diet [12].

Polyunsaturated fatty acids (PUFA) contain two or more double bonds, and are categorized into n-6 PUFA, n-3 PUFA, and n-3 long-chain polyunsaturated fatty acids (n-3 LCPUFA).

The most common one is linoleic acid (LA, 18:2), termed ‘n-6’ due to the position of the double bonds (it is present in seed oils). LA is the precursor of arachidonic acid, cannot be synthesized by the body and is therefore considered to be essential. Alpha-linolenic acid (ALA, 18:3), PUFA with double bonds in the n-3 position, is the parent fatty acid of the n-3 series and it is present found in legumes, canola oils, margarines, walnuts, and in leafy vegetables.

It is also considered essential because it cannot be synthesized by the body. Eicosapentaenoic acid (EPA, 20:5), docosahexaenoic acid (DHA, 22:6) and docosapentaenoic acid (DPA, 22:5) are found predominantly in fish-derived oils. The human body is able synthesize EPA and DHA from alpha-linolenic acid. EPA is precursor of the prostaglandins and leukotrienes. Except for the n-3 LCPUFA, recommendations (Table 8, Table 9 and Table 10) are expressed as percentage of total dietary energy (En%) or as grams per day [13].

AI is calculated by multiplying the average intake of breast milk (0.6 L/day) and the average concentration of fat, n-6 or n-3 in breast milk (40; 5.6 and 0.63 g/L respectively) and adding the median intake from complementary foods [13].

## 3. Discussion

As a consequence of prematurity, these infants generally have limited nutrient reserves since they have not been able to take advantage of the crucial last third trimester of pregnancy, period during which there is transfer and accumulation of nutrients from the mother to the fetus associated with a rapid growth. The high need for nutrients and the organ immaturity of preterm infants contribute to the difficulty of achieving dietary intakes that can allow these infants to have adequate growth [15].

Furthermore, they are often subject to physiological and metabolic stressors, such as infections, inflammation or respiratory distress, which augment their nutritional need requirements [16]. They are also at high risk of feeding problems, including delayed development of feeding skills.

When compared to milk feed, most complementary foods provide higher caloric density, and can compensate for the energy gap between increased requirements of preterm infants, and the limited caloric intake from milk feeds [17]. Reliable data on the optimal duration of milk feeds alone (breastmilk or formula) in preterm infants is not yet available [18].

Maternal breast milk (or human donor milk) is the recommended enteral nutrition for preterm or low birth weight (LBW) infants [19,20] in the first months of life. In addition to macro and micronutrients that were optimized during evolution for digestion and absorption by human infants, maternal breast milk contains numerous immuno-nutrients such as secretory immunoglobulin IgA, lactoferrin, cytokines, enzymes, growth factors, and leucocytes [21]. The nutritional requirements of preterm or LBW infants, particularly very preterm and very low birth weight (VLBW) infants, may not be met by enteral feeding with maternal breast milk alone [19], for this reason breast milk is supplemented with products called “fortifying”. Artificial formula, particularly nutrient-enriched preterm formula, might provide consistently higher levels of nutrients than maternal breast milk does, including iron and protein and has a higher caloric value [22]. Therefore, the composition and health effects of breast milk differ from those of infant formula and it may seem essential also to give different recommendations on CF to breastfed versus formula-fed infants. [2].

Indeed, several studies have demonstrated that preterm infants need a higher intake of energy, protein [23,24,25,26], long chain polyunsaturated fatty acids [26], iron [27], zinc [28], calcium and selenium [29] compared to term infants, but these indications mostly concern to the early stages of life. The study conducted by Fanaro et al. [30] showed that in almost 50% of cases, the first solid food offered to infants is low in energy density, and its protein, iron, and zinc content is negligible.

Iron is essential for brain development, and therefore iron supplementation is recommended for preterm infants until at least 6–12 months of ages [19]. By 6 months of age, the infant’s endogenous iron stores will have been used up and the need for exogenous iron increases rapidly as the physiological requirement per kg body weight becomes greater than later in life [2]. Such supplementation, upon reaching the age for weaning, could take place through foods rich in iron and not artificially.

The energy requirement also differs in relation to the severity of prematurity. Embleton et al. [31] studied dietary intake (energy 102 kcal/kg/day; protein 3.0 g/kg/day) in preterm infants (<34 weeks gestation) during the initial hospital admission. By 7 days of age, they had developed major deficits in energy (∼400 kcal/kg) and protein (13 g/kg), which were not recovered by the time of discharge; the more immature the infant the larger the deficit at discharge.

Dietary intake must also meet needs for “catch up” growth, the relation between diet and growth appears to be clear cut in that inadequate dietary intake can be directly related to poor growth. Postnatal growth retardation is very frequent during the hospital stay, with preterm infants weighing significantly less than expected at hospital discharge and often remain small throughout infancy and childhood. Nonetheless, same studies suggest that preterm infants benefit from nutritional intervention after hospital discharge, but clear-cut recommendations are difficult because the needs vary and are not well defined [32].

As the rate of weight gain increases, the incidence of cerebral palsy, Bayley Mental Developmental Index (MDI) < 70 and neurodevelopmental impairment decrease [33].

On the other hand, a high protein supply at infancy that exceeds the metabolic requirements would increase plasma and tissue concentrations of insulinogenic amino acids, the growth mediators insulin and insulin-like growth factor-1 (IGF-1), and induce a higher infant weight gain and body fat deposition as well as an increased long-term risk of obesity [34].

In the preterm, early weaning could be beneficial. However even then, the timing is often inopportune. Fanaro et al. [30] have demonstrated that weaning began before the fourth month in only 6.5% of the infants (vs. 49%) considering age from birth, and in 60.9% (vs. 95%) considering age from term.

Delayed introduction of complementary feeding may result in nutritional deficits [35,36]. Many of these problems can be prevented by starting to wean at a time when infants present readiness cues [36].

## 4. Conclusions

It is widely described in the literature that, to promote catch-up growth and neurodevelopment, preterm infants need a greater supply of energy, proteins and iron. While the contributions of micro and macronutrients in the neonatal period and in the first months of life are known, limited data are available regarding the nutritional needs that can guide the intake of food for preterm infants. Moreover, the available indications relate to the optimal time to begin the weaning, but there is a great variability among primary care pediatricians regarding the introduction of complementary foods to preterm infants [35]. The lack of firm scientific evidence that would point to the optimal age and weaning strategy result in different and often conflicting advice to the mothers [18,36,37].

Thus, it would be necessary to promote more targeted studies to better define this topic with the aim of ensuring proper growth and avoiding long-term harmful metabolic consequences in childhood or puberty, or even adulthood. Generally, premature infants begin to receive complementary foods at 7–8 months of uncorrected chronological age, or when a minimum weight of 5 kg it is reached [18]. Deferring weaning may cause nutritional deficits and delay the development of feeding skills [11].

At present time, although the need to increase the intake of some nutrients is known it is not possible to define on a scientific basis a weaning dietary scheme for preterm infants, whose nutritional needs differ from those of term babies. While waiting for the establishment of specific nutritional indications for premature infants, a valid option is represented by the use dietary reference for term infants at appropriate chronological age and possibly by modulating needs using the growth charts for preterm babies as a guide for optimal growth.

## Figures and Tables

**Table 1:** Recommended dietary intake of water.

Age	AI (Adequate Intake mL/day)
6–12 months	800 (1000)

LARN 2014 [12] e EFSA 2017 [13] and Recommended dietary intakes for Australia and New Zealand updated 2017 [14].

**Table 2:** Recommended dietary intake of protein for LARN 2014 [12].

Age	Weight	AR Average Requirement		PRI Population Reference Intakes	
Months	kg	g/kg/day	g/day	g/kg/day	g/day
6–12	8.6	1.11	9	1.32	11

Scientific evidence does not allow defining the maximum tolerable intake level (UL).

**Table 3:** Recommended dietary intake of protein for EFSA 2017 [13].

Age	AR Average Requirement	PRI Population Reference Intakes
Months	g/kg/day	g/kg/day
6	1.12	1.31
12	0.95	1.14

**Table 4:** Recommended dietary intake of protein for Australia and New Zealand, updated 2017 [14].

Age	Weight	AI Adeguate Intake	
Months	kg	g/kg/day	g/day
7–12	9	1.6	14

**Table 5:** Recommended dietary intake of carbohydrates and dietary fiber for LARN 2014 [12].

	SDT Suggested Dietary Target for the Population	AI Adequate Intake	RI Reference Intake Ranges
Total carbohydrates	Prefer food sources with low Glycemic Index (GI). Limit foods in which the reduction of GI is obtained by increasing the fructose or lipid content.	NOT AVAILABLE	45–60 En%. A minimum intake of 2 g/kg/day is sufficient to prevent ketosis. Upper limit of 65 En% it can be accepted in conditions of high energy expenditure, from intense physical activity
Sugars (sugars naturally present in milk, fruit and vegetables and added sugars)	Limit the consumption of sugars to <15 En%. An intake >25 En% (95th percentile of introduction into the Italian diet) is to be considered potentially linked to adverse health events (increase in triglycerides and cholesterol, and impaired response to glucose and insulin). Limit the use of fructose as a sweetener. Limit the use of foods and drinks formulated with fructose or high fructose corn syrups.	NOT AVAILABLE	NOT AVAILABLE
Dietary Fiber	Prefer foods naturally rich in dietary fiber, such as whole grains, legumes, fruits and vegetables.	>1 year: 8.4 g/1000 cal (2 g/MJ)	NOT AVAILABLE

En%: percentage of the total energy of the diet.

**Table 6:** Recommended dietary intake of carbohydrates and dietary fiber for EFSA [13].

Age (Years)	Total Carbohydrates	Dietary Fibre
1	45–60 En%	10 g/day

En%: percentage of the total energy of the diet. EFSA does not report any references relating to the age of 6–12 months.

**Table 7:** Recommended intake of carbohydrates and dietary fiber for Australia and New Zealand [14].

Age (Months)	AI Adeguate Intake
7–12	95 g/day

AI is based on an average volume of 0.60 L/day milk at 74 g/L (44 g/day), plus an amount from complementary foods of 51 g/day [13].

**Table 8:** Recommended dietary intake of fats for LARN 2014 [12].

**Age** **6–12 Months**		**SDT** **Suggested Dietary Target for the Population**	**AI** **Adequate Intake**	**RI** **Reference Intake Ranges**
	Total lipids		40 En%	
	Saturated fatty acids	<10 En%		
	PUFA			5–10 En%
	PUFA n-6			4–8 En%
	PUFA n-3		EPA-DHA 250 mg + DHA 100 mg	0.5–2 En%
	Trans fatty acids	As little as possible		

En%: percentage of the total energy of the diet; PUFA: polyunsaturated fatty acids; PUFA n-6: polyunsaturated fatty acids of the n-6 series; PUFA n-3: polyunsaturated fatty acids of the n-3 series; EPA: eicosapentaenoic acid; DHA: docosahexaenoic acid.

**Table 9:** Recommended dietary intake of fats for EFSA 2017 [13].

**Age** **6–12 Months**		**SDT** **Suggested Dietary Target for the Population**	**AI** **Adequate Intake**	**RI** **Reference Intake Ranges**
	Total lipids			40 En%
	Saturated fatty acids	As little as possible		
	Linoleic		4 En%	
	α-linoleic		0.5 En%	
	*DHA*		100 mg/die	
	Trans fatty acids	As little as possible		

Docosahexaenoic acid (DHA) intakes at levels of 50 to 100 mg per day are sufficient for visual function in the complementary feeding period [13].

**Table 10:** Recommended dietary intake of fats for Australia and New Zealand updated 2017 [14].

**Age** **7–12 Months**		**AI** **Adequate Intake** **g/day**
	Total lipids	30
	n-6 PUFA	4.6
	n-3 PUFA	0.5
